# Cronos: A Machine Learning Pipeline for Description and Predictive Modeling of Microbial Communities Over Time

**DOI:** 10.3389/fbinf.2022.866902

**Published:** 2022-08-09

**Authors:** Aristeidis Litos, Evangelia Intze, Pavlos Pavlidis, Ilias Lagkouvardos

**Affiliations:** ^1^ School of Medicine, University of Crete, Heraklion, Greece; ^2^ Institute of Computer Science, Foundation of Research and Technology, Heraklion, Greece; ^3^ School of Science and Technology, Hellenic Open University, Patras, Greece; ^4^ Core Facility Microbiome—ZIEL Institute for Food and Health, Technical University of Munich, Freising, Germany

**Keywords:** microbial profiles, microbiome, machine learning, De novo clustering, microbial communities, infant gut maturation, multinomial logistic regression, time-series

## Abstract

Microbial time-series analysis, typically, examines the abundances of individual taxa over time and attempts to assign etiology to observed patterns. This approach assumes homogeneous groups in terms of profiles and response to external effectors. These assumptions are not always fulfilled, especially in complex natural systems, like the microbiome of the human gut. It is actually established that humans with otherwise the same demographic or dietary backgrounds can have distinct microbial profiles. We suggest an alternative approach to the analysis of microbial time-series, based on the following premises: 1) microbial communities are organized in distinct clusters of similar composition at any time point, 2) these intrinsic subsets of communities could have different responses to the same external effects, and 3) the fate of the communities is largely deterministic given the same external conditions. Therefore, tracking the transition of communities, rather than individual taxa, across these states, can enhance our understanding of the ecological processes and allow the prediction of future states, by incorporating applied effects. We implement these ideas into Cronos, an analytical pipeline written in R. Cronos’ inputs are a microbial composition table (e.g., OTU table), their phylogenetic relations as a tree, and the associated metadata. Cronos detects the intrinsic microbial profile clusters on all time points, describes them in terms of composition, and records the transitions between them. Cluster assignments, combined with the provided metadata, are used to model the transitions and predict samples’ fate under various effects. We applied Cronos to available data from growing infants’ gut microbiomes, and we observe two distinct trajectories corresponding to breastfed and formula-fed infants that eventually converge to profiles resembling those of mature individuals. Cronos is freely available at https://github.com/Lagkouvardos/Cronos.

## 1 Introduction

Advances in sequencing technologies allowed the investigation of diverse environments in terms of bacterial community structure as standardized practice (Mukherjee et al., 2021). Studies of microbial communities over time are steadily gaining in popularity compared with the majority of studies, in which a single time point is investigated, allowing for a further understanding of community dynamics.

Microbial communities consist of multiple species entangled in complex interactions that affect their individual behavior, overall system dynamics, and environmental niche properties (Stubbendieck et al., 2016). Internal phenomena include direct interactions, such as mutualism (Morris et al., 2013) or competition (Stubbendieck et al., 2016) and indirect interactions, such as quorum sensing (Miller and Bassler, 2001). Internal interactions in combination with external factors, such as antibiotics (Iizumi et al., 2017), infants’ birth mode, or diet (Kim et al., 2019), affect the individual bacteria behavior and shape the environment landscape (Tan et al., 2021). Therefore, a complete understanding of microbial systems can only be achieved by studying the overall microbial communities rather than each microbial organism in isolation.

Time-series analysis of abundance and co-occurrence of microbes have been investigated mainly via traditional statistical methods (Chaffron et al., 2010; Steele et al., 2011). Several bioinformatic tools for bacterial time-series analysis have been developed, exploiting the increasing data availability. These tools, along with other studies, focus mainly on single or specific taxa and their relative abundance over time (Vergin et al., 2013; Sharon et al., 2013; Xia et al., 2011; Ki et al., 2018; Zhang et al., 2019). However, those approaches inherit the limitations and assumptions of the statistical methods used. Relying on experimental design labels may mask distinct patterns or structures in each group and therefore misinterpret the microbial community trajectories. Often, abundance values for a given group of samples at a time point can exhibit multiple modes implying the existence of more than one underlying distribution. Comparing values among time points with statistical methods relying on means or ranks is not appropriate for multimodal datasets.

In the first 2 years of life, the gut microbiome is subjected to many compositional changes (Bäckhed et al., 2015; Stewart et al., 2018). The procedure toward the adult microbiome is often called maturation (Mesa et al., 2020). Evidence suggests an association between infant gut bacteria and diet (Pannaraj et al., 2017; Jiang et al., 2018; Camacho-Morales et al., 2021), the way the infant was delivered (Jakobsson et al., 2014), antibiotic usage (Korpela et al., 2020; Lemas et al., 2016), maternal body mass index (Soderborg et al., 2018), or even environmental factors (Sugino et al., 2021). Alterations of the human gut microbiome during the maturation procedure motivate the analysis of microbiome profiles using time-series approaches.

In this study, we propose a novel framework for microbial community time-series data analysis. Embedded in an R-based tool, Cronos, is based on the following premises and concepts. Intrinsic microbial community structures within a time point are shaped due to specific attractor states (Estrela et al., 2022; Goldford et al., 2018). These states can be identified by unsupervised machine learning techniques. Microbial communities’ evolution can be explored by capturing transitions among attractor states over time. We developed an implementation of this concept in Cronos software. Cronos applies machine learning techniques to analyze complete microbial profiles over time and describe the attractor states (Costea et al., 2018). Our software explores the microbial community profile evolution by capturing transitions among clusters over time. As a consequence, it is able to predict future community structure states. Cronos is freely available, as an open-source code at https://github.com/Lagkouvardos/Cronos.

## 2 Materials and Methods

Cronos is an R script that performs the tasks of 1) dividing and labeling the samples based on the time points, 2) calculating the pairwise UniFrac distances among the samples at every time point, 3) performing *de novo* clustering of the samples profiles, 4) calculating and visualizing the taxonomic representation of clusters, 5) applying Markovian property test, 6) transition modeling based on given metadata, and 7) predicting future states.

Cronos functions rely on R packages ade4, dplyr, GUniFrac, phangorn, cluster, fpc, markovchain, spgs, caret, nnet, gtools, mclust, igraph, and network, which Cronos installs automatically if required, along with all of their dependencies. Cronos requires three files as inputs. A table of microbial profiles (e.g., OTU or ASV abundance tables), a mapping file containing information about the time points and the corresponding metadata of the samples, and a phylogenetic tree of all taxa in the profiles table.

### 2.1 *De novo* Clustering, Evaluation, and Validation

Cronos calculates the GUniFrac, a beta-diversity distance metric variant (Chen et al., 2012) of the UniFrac distance methods (Lozupone and Knight, 2005), for each pair of samples at every time point, using the phylogenetic tree input, to create a dissimilarity matrix. Then, *de novo* clustering is performed via the partitioning around medoid (PAM) method (Schubert and Rousseeuw, 2021; Costea et al., 2018). Cronos assesses the optimal number of clusters via the Calinski–Harabasz index.

Cronos applies a brute force method to select the optimal number of clusters at every time point. Clustering via PAM is performed using as the number of clusters (k) all the numbers between two and nine. Due to computational constraints, the maximum number of clusters was set to nine. The optimal number of clusters is assessed using the Calinski–Harabasz index (Calinski and Harabasz, 1974) also known as the variance ratio criterion, from the fpc R package. The Calinski–Harabasz index is translated into the ratio of the sum of between clusters dispersion to intercluster dispersion. Higher Calinski–Harabasz index values indicate better clustering performance.

Calinski–Harabasz (s) index is calculated as

$$s=\left(\frac{tr\left(Bk\right)}{tr\left(Wk\right)}\;*\;\frac{n-k}{k-1}\right)$$ (1)

where n is the sample size divided into k clusters, tr (Bk) is the trace of the between cluster dispersion matrix, and tr (Wk) is the trace of the within-cluster dispersion matrix defined by

$$Wk=\overset{k}{\underset{p=1}{\sum }}\underset{x\in C_{p}}{\sum }\left(x-C_{p}\right){\left(x-C_{p}\right)}^{T}$$ (2)

$$Bk=\overset{k}{\underset{p=1}{\sum }}n_{p}\left(C_{p}-C_{E}\right){\left(C_{p}-C_{E}\right)}^{T}$$ (3)

where C_p_ is the set of points in cluster p, C_E_ the center of cluster E, and n_p_ the number of points in cluster p.

In order to achieve high clustering resolution but avoid overclustering, we determined the optimal number of clusters based on two rules: The maximum consecutive Calinski–Harabasz score difference and the difference between the absolute maximum of Calinski–Harabasz scores and the one with the highest difference. Such an approach, empirically, demonstrated both high clustering resolution and avoided meaningless overclustering.

First, we calculate the Calinski–Harabasz indexes for two to nine clusters. Second, we calculate the difference between Calinski–Harabasz indexes for every two consecutive numbers of clusters and select the highest. Third, we calculate the difference in Calinski–Harabasz scores between the preselected and the absolute maximum of CH scores.

$$k=\left\{\begin{array}{c} argmax\left(S_{k}\right)\;\;\;ifmaxS_{k}-maxS_{argmax\left(S_{k}-S_{k+1}\right)}\;\geq \left|\max\left(S_{k}-S_{k+1}\right)\right|\;\\ argmax\left(S_{k}-S_{k+1}\right)\;\;ifmaxS_{k}-maxS_{argmax\left(S_{k}-S_{k+1}\right)}\;<\left|\max\left(S_{k}-S_{k+1}\right)\right|\; \end{array}\right.\left(\right)$$ (4)

The optimal number of clusters is selected as the absolute maximum of Calinski–Harabasz scores if $maxS_{k}-maxS_{argmax\left(S_{k}-S_{k+1}\right)}\geq |\max\left(S_{k}-S_{k+1}\right)|$ or the preselected k $maxS_{k}-maxS_{argmax\left(S_{k}-S_{k+1}\right)}<|\max\left(S_{k}-S_{k+1}\right)|$ .

**FIGURE 1:**
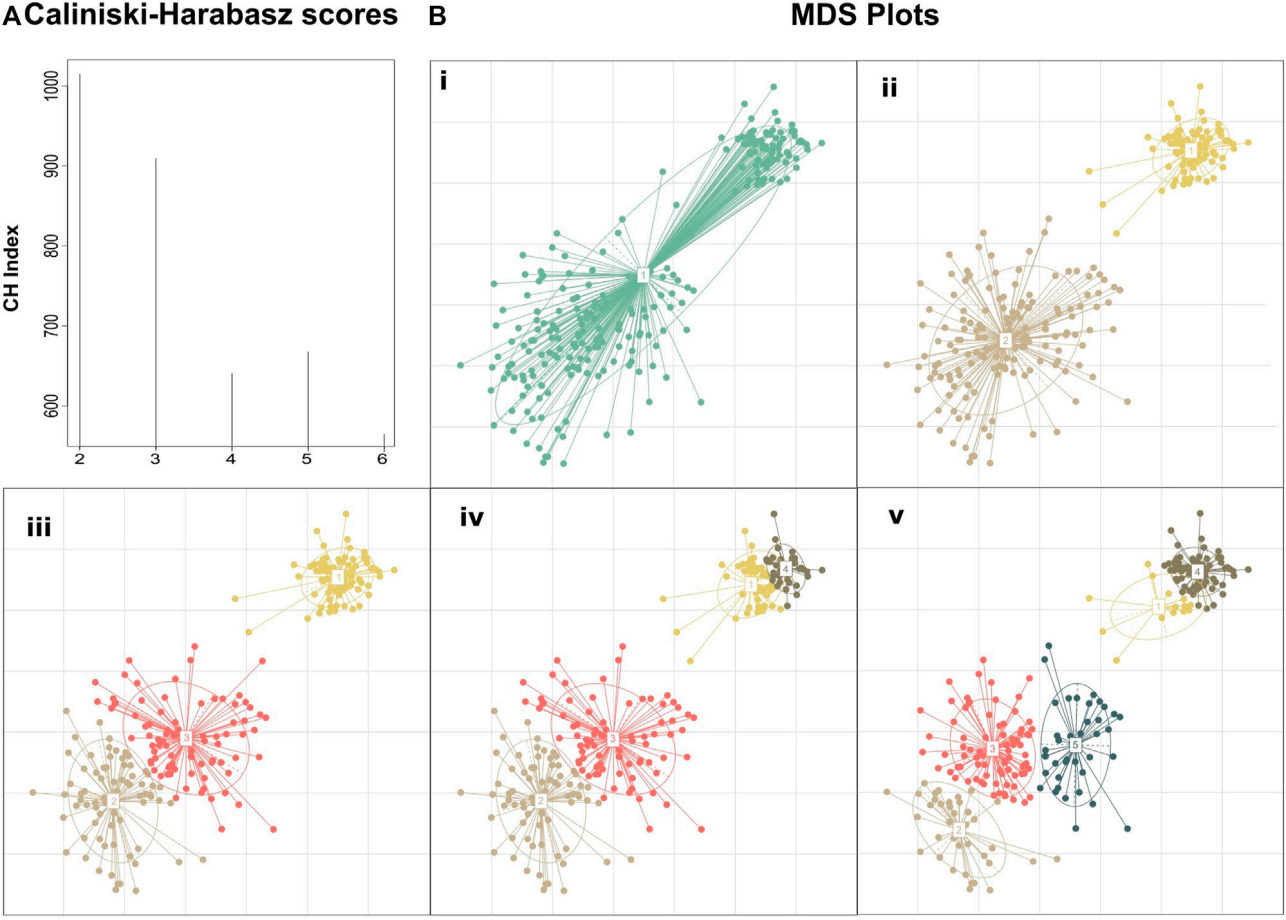
Clustering performed on a manually created dataset demonstrating the added insight on the structure achieved by selecting the number of clusters based on clustering quality drop rather than simply using the clustering with the maximum score. **(A)** Calinski–Harabasz indexes calculated for PAM clustering with k ranging from 2 to 6. Although k = 2 shows the highest score suggesting the existence of two primary groupings in the dataset, k = 3 also fits well into the dataset, revealing the composite nature of the second cluster. Further subclustering results in a large drop in the clustering quality suggesting that the dataset does not fit well to the number of clusters **(B)** MDS plots of the dataset at different *de novo* clustering levels (i) MDS plot of the unclustered dataset, (ii–v) PAM clustering of the dataset for k = 2–5.

The motivation behind this approach is that if we rely only on the maximum CH score, we will detect just a crude clustering of the data, overlooking, thus, any fine data clustering (Figure 1). By assessing the value of k by the Eq (4), we will obtain the highest possible resolution on a given time point (any further refinement will diminish the clustering quality) while keeping the CH score of clustering close to the absolute maximum score. To highlight this approach we created a hypothetical dataset manually derived from three Gaussian distributions with standard deviations of 0.1, 0.4, and 0.6 and means (6.5,6.5), (3,3), and (4,4), respectively. The absolute maximum Calinski–Harabasz value indicates that the optimal number of clusters for this dataset is 2, even though we manufactured the dataset from three different Gaussian distributions (Figure 1).

Since PAM clustering will divide the dataset into at least two groups even when data contain no clusters, Cronos also performs a validity check of clustering. To address this issue, we apply a Bayesian information criterion (BIC)-based methodology to evaluate whether k clusters (k > 1) are better than a scenario with no clusters for each time point. We apply Gaussian mixture model (GMM) clustering with 1 and the optimal number k of clusters as components, using the mclust R package. To compare the two clustering outcomes from GMM, the BIC score was calculated using the same R package.

### 2.2 Transition Analysis

Clustering at each timepoint results in the characterization of samples over time. To further understand the evolution of the microbiome profiles, Cronos primarily checks for the Markovian property of the transitions of clusters from each time point to the next. A transition acquires the Markovian property when it depends only on the current state and not on any previous one. A custom test was created to verify the first-order Markovian assumption (i.e., future state does not depend on the exact previous one but only the current) among the transitions of all samples based on the verifyMarkovProperty test of markovchain R package. The test examines all successive triplets of time points, in terms of states–cluster assignments. Let x_1_, x_2_, …, x_N_ be a set of observations with N the optimal number of clusters selected and n_ijk_ is the number of times t (1 ≤ *t* ≤ *N* − 2) such that x_t_ = i, x_t+1_ = j, x_t+2_ = k; then, if the Markov property holds, n_ijk_ follows a Binomial distribution with parameters n_ij_ and p_jk_.

A classical chi-square test can check this distributional assumption, since

$$\underset{i}{\sum }\underset{j}{\sum }\underset{k}{\sum }\frac{{\left(n_{\mathrm{ijk}}-n_{ij}p_{jk}\right)}^{2}}{n_{ij}p_{jk}}∼\chi^{2}\left(d\right)$$ (5)

where d is the number of degrees of freedom. The number of degrees of freedom d of the chi-square distribution is given by d = r − q + s − 1, where s denotes the number of states i in the state space such that n_i_ > 0, q denotes the number of pairs (i, j) for which n_ij_ > 0, and r denotes the number of triplets (i, j, k) for which n_ijk_ > 0.

### 2.3 Transition Modeling

Cronos models the states at each time point (response variable) as a function of the metadata at this time point and the state at a previous time point (explanatory variables) by applying multinomial logistic regression via the multinom function of the nnet R package. For each time point, we create a matrix of explanatory variables using the cluster label on a given time point and the metadata as columns and the samples as rows.

To evaluate the predictions, Cronos divides the dataset into training and test sets using two different methods. First, we apply a leave one out (LOO) procedure, where all the dataset is used to train the model except one sample, which is used as the test set. The second method refers to stratified splits, which is performed via the createDataPartition function of the caret R package and splits the dataset into train and test sets with the same ratio of samples per label.

Cronos evaluates the accuracy of classification as the percentage of correct predictions that the model made:

$$A=\frac{correctPredictions}{N}$$ (6)

where N is the number of samples on the set and returns the mean accuracy over a prespecified number of iterations for both the training and the test sets, all the division methods, and all the time points used to create the models. Mean accuracy of a model is calculated as follows:

$$Acc=\frac{1}{T}\overset{T}{\underset{i=1}{\sum }}\frac{correctPredictions}{N}$$ (7)

where N is the number of samples on the set and T is the number of iterations. Partitions with the LOO method are iterated over all samples, whereas the stratified splits method assigns samples on the test set ensuring that the train and test sets have approximately the same percentage of samples of each target class as the complete set.

**FIGURE 2:**
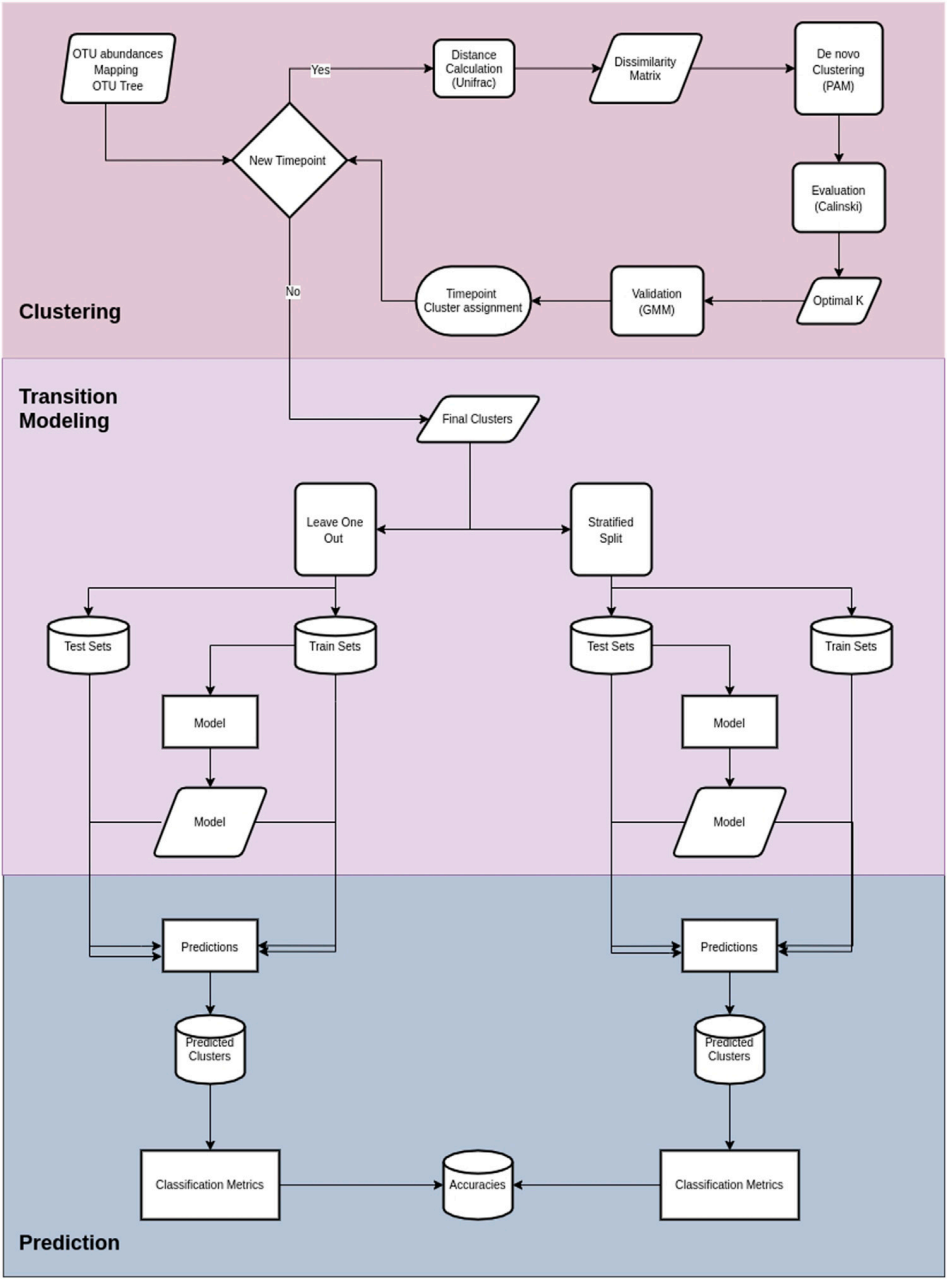
Cronos’ pipeline. The first section illustrates the procedures on Cronos from obtaining the data to forming complete clustering assignments.The middle section demonstrates the modeling of transitions from clusters on a time point to any later. The third section displays the prediction procedure and classification metrics.

Cronos performs classification to predict the cluster on all time points but the first, with both partitioning methods for all the possible combinations of metadata provided, combined with cluster assignment, including models without metadata, both for the training and test sets. The classification performance of Cronos is compared to the random classifier, which labels all the possible outcomes of the predicted variable with the same frequency. Cronos’ complete pipeline is shown in Figure 2.

### 2.4 Cluster Representation

Every cluster of microbial profiles is represented via its medoid. Cronos describes every medoid composition at all taxonomic levels above the genus to provide further insight into its community structure via binning (cumulative abundance of all OTUs/ASVs belonging to the same taxon). Furthermore, the profiles are illustrated as barplots. To enhance the visualizations, there is an option to agglomerate low abundance taxa into the category called “Others” using a selected by the user threshold (default 5%).

### 2.5 Case Study

Cronos was tested on the fecal microbiome data from a study investigating the effects of formula milk and breastfeeding on infants’ gut microbiome over the span of 2 years (Bazanella et al., 2017). The dataset consists of 106 infants from the Munich region with samples taken over 1, 3, 5, 7, 9, 12, and 24 months of age. Information on the mode of delivery (vaginal or Cesarean) was available and taken into account in our analysis. In addition to the infant data, we used as a reference for matured gut microbiome the sequence data from the stool samples from 216 healthy lean students of the Technical University of Munich. None of the students had been taking antibiotics in the last 3 months, had any known diseases, or were on long-term medication. The preprocessing of the raw data was performed with the IMNGS platform (Lagkouvardos et al., 2016) implementing the UNOISE version 3 (Edgar, 2016) and UPARSE (Edgar, 2013) pipelines, using the default parameters. The primary analysis outputs were used as inputs in Cronos. The raw data of the two studies are publically available at European Nucleotide Archive under accessions PRJEB21196 and PRJEB47555.

## 3 Results

We applied Cronos to the data retrieved from the infant study of Bazanella et al. (2017) combined with the healthy students reference dataset. The samples were characterized in terms of OTU abundance via the IMNGS platform; the outputs were used as direct input for the Cronos tool.

### 3.1 *De novo* Profile Clustering

The Calinski–Harabasz indexes calculated for each clustering procedure are graphically demonstrated and stored automatically using Cronos (Supplementary Figure S1). Cronos’ automated method for selection of the optimal number of the *de novo* clusters suggested that partitioning the data into two or three groups reflects the intrinsic organization of the microbial profiles of the infants at each time point and of the students used as an external reference (Table 1).

**TABLE 1:** Optimal number of clusters selected automatically in Cronos for all time points. The first row represents the time point in months of age, whereas the second shows the different number of similar microbiome profiles.

Time point (Months of age)	1	3	5	7	9	12	24	References
Optimal Number of Clusters	2	3	2	2	3	3	2	3

### 3.2 Maturation Process

**FIGURE 3:**
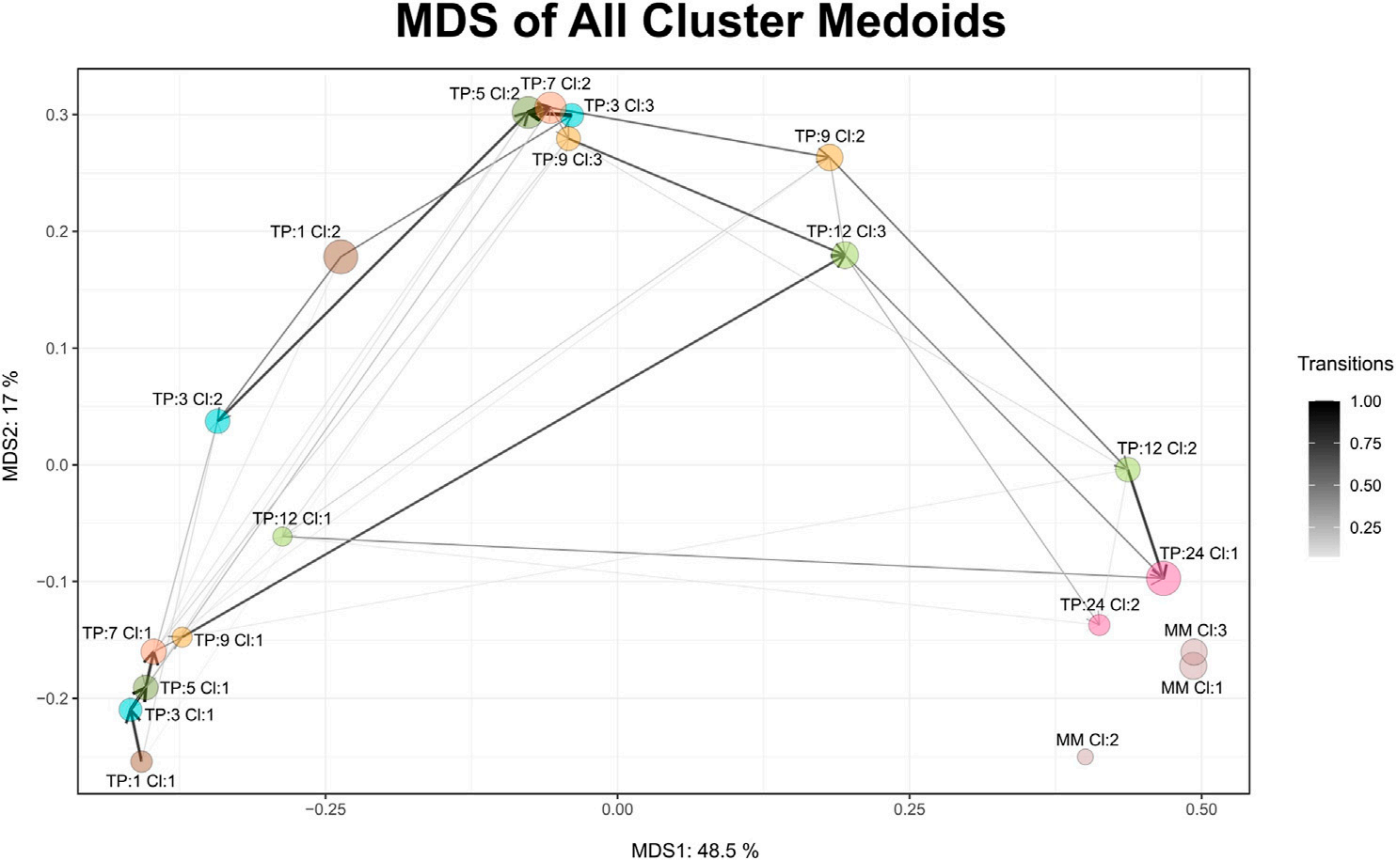
MDS graph of cluster medoids of all clusters on all time points and the external reference as produced by Cronos. TP represents the time point and CL the cluster. MM abbreviation stands for mature microbiome and refers to the external reference samples. Circle size is relative to the percentage of the samples found to belong in the cluster, whereas the connecting arrows are directed from the earliest to the latest time point and their width is relative to the percentage of the samples following the transition.

Maturation, as a time-dependent process, is illustrated in Cronos via an MDS plot of all cluster medoids, to compare the relative distances between clusters within the dataset and any external reference time point given. Every microbiome profile cluster is represented by its medoid. The evolution trajectory of the microbiome over time is demonstrated by connecting the medoids as shown in Figure 3.

Microbiome profiles of 24 months of age children are relatively close to the adult external references, whereas early life clusters occur closer to each other, highlighting the maturation process. Three main areas of microbiome profile similarity are shown in the graph. The first, on the bottom left side, contains almost half of the early life clusters, dominated by breastfed infants. The top center one contains almost the other half of early life clusters and the bottom right one holds the external reference and 2-year-old clusters. The average distance of infant clusters on all time points compared to the external reference clusters of students decreases as the infants age (Supplementary Figure S5), emphasizing the maturation process, as older infants have microbial profiles relatively closer to the adult students.

### 3.3 Sample Transitions Through Time

**FIGURE 4:**
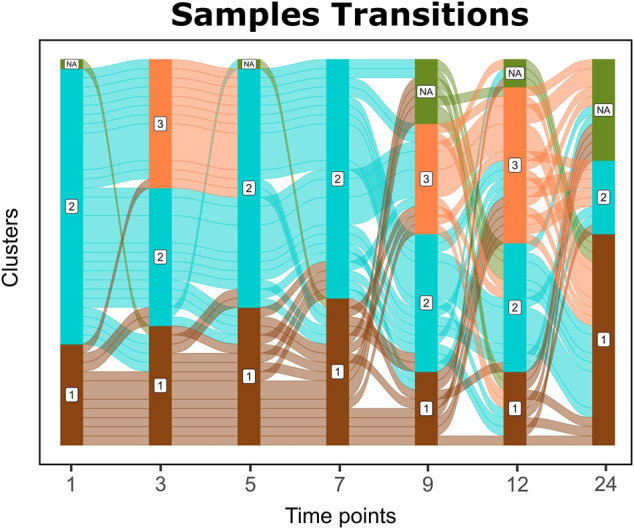
Alluvial graph of sample transitions between clusters over time. NAs represent nonavailable data for the corresponding time point, whereas 1, 2, and 3 represent the cluster on the corresponding time point.

Sample transitions between clusters over time are visualized in Cronos via Alluvial graphs (Figure 4). For the first months and until the seventh month of age, infants’ profiles show common transition patterns switching largely in unison among the time point clusters. At later time points, the infants’ microbiome endures many changes in terms of composition, illustrated by cluster alterations (samples entangled between clusters) on consecutive time points. As the infants age, their microbiome profiles tend to converge toward the adult reference. Longer periods between sampling and the introduction of a third cluster on 9 and 12-month-old children might explain the increase in sample transitions between clusters during these stages.

### 3.4 Cluster Representation

Every cluster is represented by its medoid. Cronos’ automated pipeline describes and illustrates the microbial composition of all cluster medoids on all taxonomic levels above genus (Supplementary Tables S1, S2, S3). The representation of all clusters on a family level is shown in Figure 3 (Supplementary Figures S3, S4 on Order and Class levels).

**FIGURE 5:**
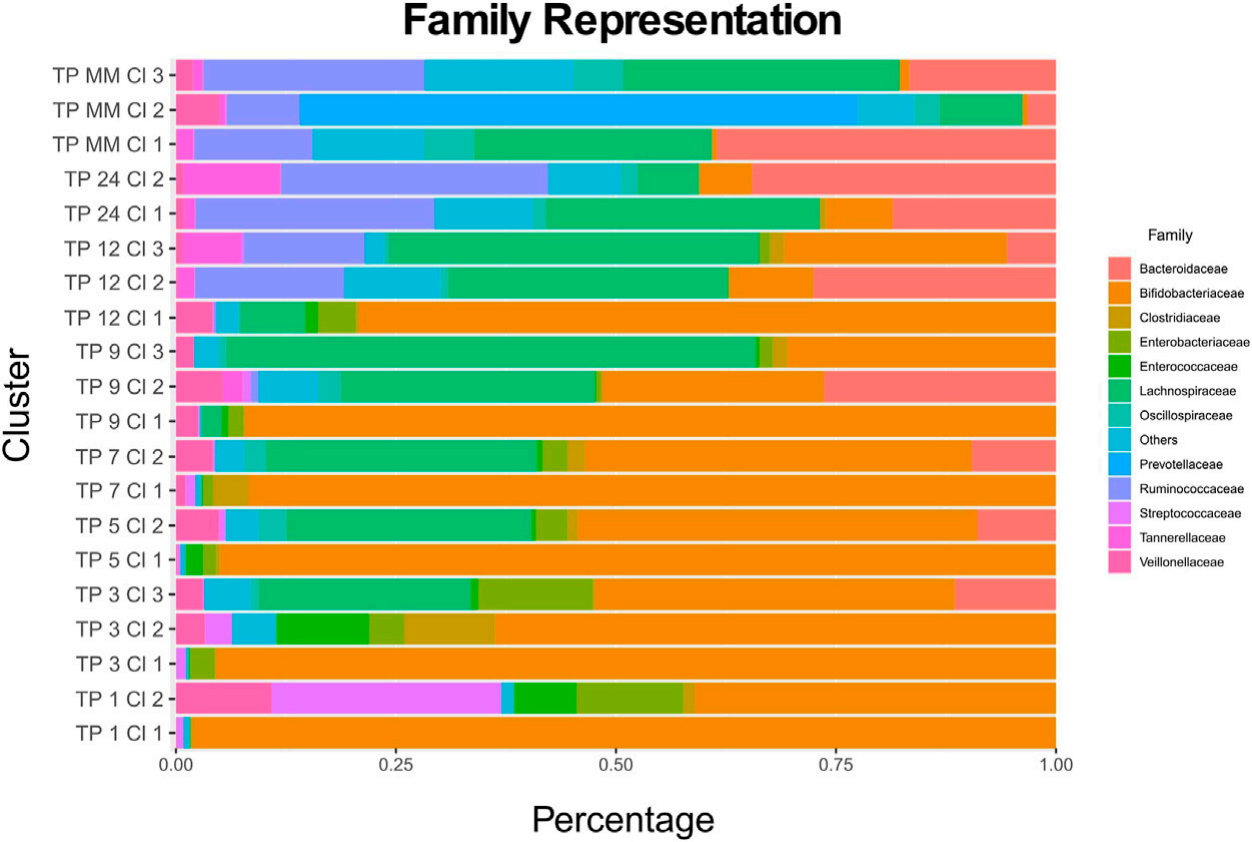
Every cluster composition at a family level. TP stands for time point and Cl for the cluster. Families with an abundance lower than 5% across all medoids are cumulatively shown as Others.

The relative distances of cluster profiles can be shown even at a family level, highlighting the importance of a beta-diversity distance metric and the final number of cluster decisions. Clusters of 1-month-old infants are highly associated with the two types of diet. TP1-CL1 contains significantly more breastfed infants than expected (one-sided *x* ^2^ test *p* = 0.00035), whereas TP1-CL2 contains more than expected formula-fed infants (one-sided *x* ^2^ test *p* = 0.03069). TP1-CL1 is dominated by the *Bifidobacteriaceae* family, whereas TP1-CL2 has a more diverse profile, with lower *Bifidobacteriaceae* and higher *Streptococcaceae* and *Enterobacteriaceae* abundances (Figure 5). Clusters of 3, 5, and 7 months of age have similar compositions (Figure 5), reflected as close relative distances in the multidimensional scaling projection (MDS plot, Figure 3). The majority of 9- and 12-month-old infants’ profiles start diverging. TP9-CL1 and TP12-CL1 represent late immature profiles, where the *Bifidobacteriaceae* family dominates. TP9-CL2 and TP12-CL2 show an increase in *Bacteroidaceae* family abundance, whereas TP9-CL3 and TP12-CL3 have a higher abundance of the *Lachnospiraceae* family (Figure 5). Microbial profiles of 2-year-old infants separate into two clusters, where the feeding groups co-occur. Thus, there is no association between the two types of diet and microbial profile clustering for any of the two clusters (one-sided *x* ^2^ test *p* = 0.65 and 0.45, respectively). TP24-CL1 and TP24-CL2 are characterized by higher *Bacteroidaceae* and *Lachnospiraceae* abundances, respectively, whereas both contain a sizable proportion of *Ruminococcaceae* (20%). Clusters of 2-year-old infants are relatively closer to the reference profiles of mature individuals. The reference group is partitioned into three clusters that resemble the described enterotypes with MM-CL1 being the “*Bacteroides*” group, MM-CL2 the “*Prevotella*” and MM-CL3 the “*Ruminococcus*” group (Arumugam et al., 2011).

### 3.5 Transition Modeling

**FIGURE 6:**
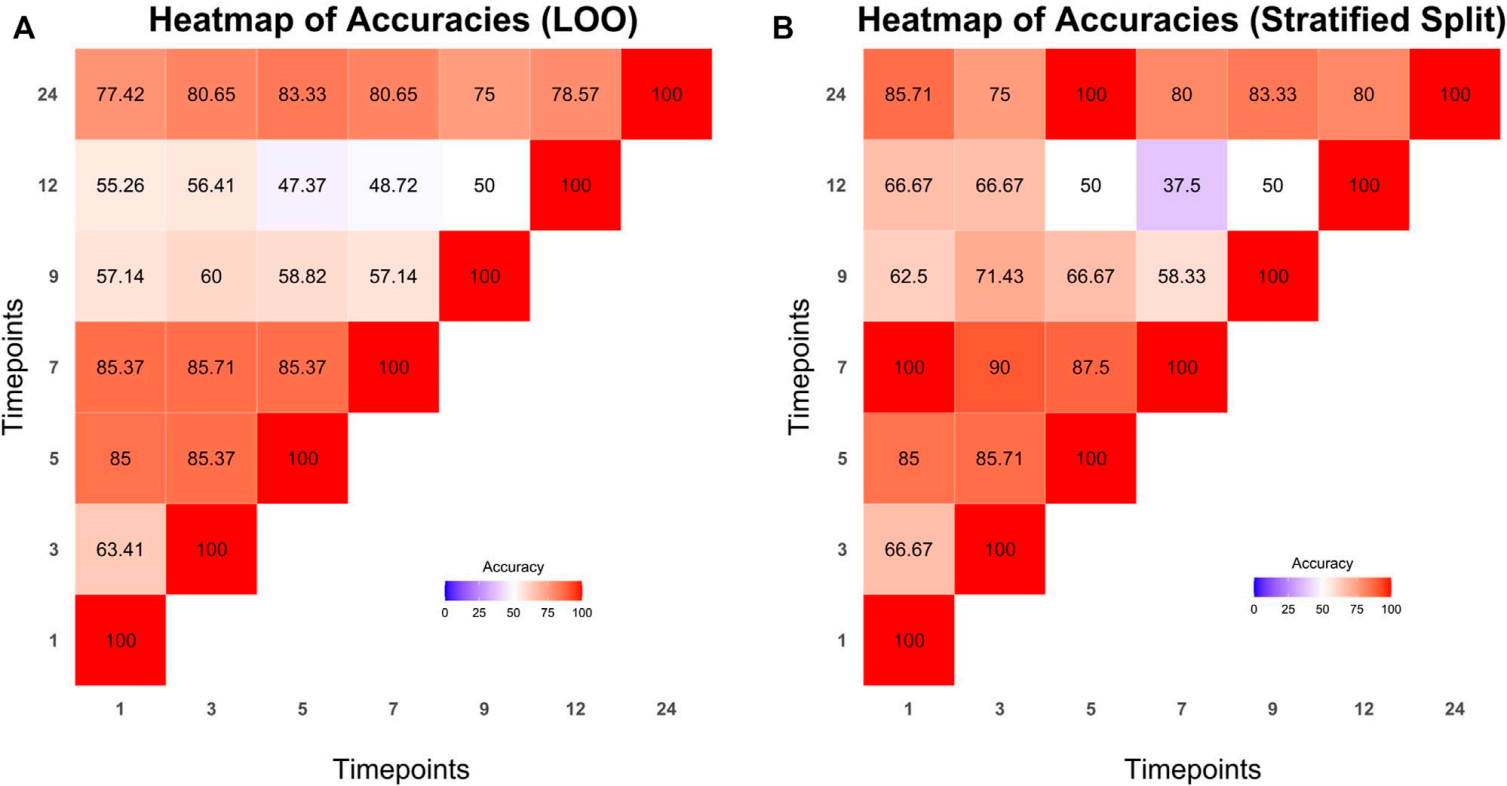
Heatmaps presenting the prediction accuracies achieved from leave one out **(A)** and stratified splits methods **(B)** respectively. Time points from which predictions are made are shown on the *x* axis while predicting time points are represented on the *y* axis.

The dataset was split into train and test sets with the aforementioned methods (LOO and stratified splits). Microbiome profile transitions between clusters on different time points of all possible train sets were modeled by Cronos via multinomial logistic regression. Furthermore, using the model created by the training sets, Cronos predicted the clusters on all time points of the samples based on the provided matrix with metadata. Prediction performance was evaluated via the accuracy metric. The achieved accuracies are visualized in Cronos with multiple barplots according to the predicting and explanatory time point. Moreover, Cronos’ automated pipeline creates heatmaps for both splitting methods (Figure 6).

All the predictions made by Cronos are compared to a trivial classifier, the random one, where the probability of all clusters is equal (i.e., 1/N where N is the number of clusters Supplementary Tables S4, S5 show the comparison of the highest accuracies achieved from models with LOO and stratified splits methods to the trivial random classifiers into the test sets).

## 4 Discussion

### 4.1 *De novo* Clustering and Cluster Validation

We apply a “Zoom out” methodology by assessing every sample as its whole microbial profile, rather than individual taxa. Cronos’ automated pipeline incorporates the beta-diversity distance between samples by exploiting the advantages of the GUniFrac distance metric. Dirichlet multinomial mixtures (Holmes et al., 2012) widely used on microbiome data (Hosoda et al., 2020; Subedi et al., 2020) assume a prior distribution and are based on the abundances. Here, *de novo* clusters reflect the profile distance between samples adding another layer of information. For the clustering of the samples, we apply the partitioning around medoids algorithm, which allows us to represent every cluster by its medoid. This method has been successfully applied in studies spanning from the gut (Stokholm et al., 2018; Khine et al., 2019; Lee et al., 2020) to saliva (Acharya et al., 2017) microbiome.

*De novo* clustering is applied to all time points separately to specify the exact stages and future transitions of the microbial profiles. The maturation process through clustering has been well established (Stewart et al., 2018; de Muinck and Trosvik, 2018), whereas the divergence in specific time points remains unexplored. Here, by dividing the dataset into time points and applying clustering procedures to all, we provide a deeper understanding of microbial profile divergence.

A novel approach is incorporated to effectively divide the samples at a time point into clusters of a similar microbial profile, based on the GMM clustering algorithm (Pasarkar et al., 2021; Zhang et al., 2017). We compare clustering results for the optimal number of clusters to 1 as GMM components, in order to examine whether the data effectively separate.

### 4.2 Transitions Through Time and Modeling

Exploring the sample transitions between clusters at different time points enables the understanding of the effectors that shape a microbial profile’s fate. Many machine learning techniques have been applied to microbiome data (Marcos-Zambrano et al., 2021). Cronos operates under the assumption that minor compositional differences among the members of a certain cluster of profiles are less important when the fate of the community as a whole is examined. When this assumption is not fulfilled and the presence or absence of taxa with little contribution to the overall cluster assignment determines the future of the community structure, the accuracy of the method might be low. The selection of cluster assignment rather than taxa abundances, and the introduction of metadata results in a small number of explanatory features. Due to the low number of features and interpretability losses that come with high complexity classification algorithms (Marcos-Zambrano et al., 2021), we select multinomial logistic regression, a method widely used on microbiome data (Kaszubinski et al., 2020; Lundgren et al., 2018; Xia et al., 2013) to model the transitions between clusters on different time points.

The importance of features on microbial profile fate is translated as predictability. Features or combinations of features that can better interpret cluster assignment on predicting time points are deemed to be the most important in the development of the microbiome profile in the time between examining and predicting time points. Cronos models for every possible transition and possible mixture of features to fully reflect the predictability of features on all combinations of timepoints and overall, aiming to detect the best time for interventions to steer a microbial profile’s fate. Every model designed in Cronos is compared to the trivial random classifier that predicts all classes with equal probability.

### 4.3 Maturation

Our findings are in accordance with the well-documented microbiome patterns of early life. Breastfed infant profiles consist, mainly, of *Bifidobacteriaceae* family members, whereas formula-fed infants show higher diversity, colonized earlier by *Enterobacteriaceae*, *Bacteroidaceae*, and *Lachnospiraceae* members (Milani et al., 2017; Fallani et al., 2011; Koenig et al., 2011). Furthermore, our analysis, captures the decrease in *Bifidobacteriaceae* and the gradual increase of *Ruminococcaceae*, *Lachnospiraceae*, and *Bacteroidaceae* relative abundances, after the introduction of solid food, until the second year of life as established before (Laursen et al., 2016; Fallani et al., 2011). Cronos provides comparisons of taxonomic composition for the cluster medoids as a proxy of the corresponding cluster. The statistical comparisons of similar profiles fall outside of the scope of the tool. Therefore, using the outputs of Cronos, external tools like Rhea (Lagkouvardos et al., 2017) or QIIME (Caporaso et al., 2010) can easily perform these statistical comparisons of taxa among clusters, considering all their constituting members.

## 5 Applications and Future Work

Cronos is a bioinformatic tool that could also be used for other types of environments where bacterial communities dominate, such as soil or marine over the course of the year or several years, aiming to understand the microbiome progression or the suitable response to direct the microbial composition of the environment. Uses of Cronos extend from natural environments to man-made environments, such as open pond bioreactors. Possible uses might also include human gut microbiome over the progression of diseases, sampling over different stages of the disease, aiming to discover the proper antibiotic response or microbiome role in disease progression and phenotype.

For further understanding of infant gut microbiome profiles, more data are required, since the dataset used here as a case study was obtained from a limited geographical region and thus may not include all the possible states. Greater sample size could furthermore benefit the prediction of future states by training a model with more samples.

In future versions of Cronos, we want to include more classification techniques, such as random forest and support vector machines to acquire models that could enhance our transition description. In addition, we would like to introduce further classification performance metrics, such as precision, recall, and F1-score in order to represent model prediction performance extensively. Moreover, we would like to add further clustering performance metrics, such as the Akaike information criterion and silhouette coefficient to further describe cluster divergence.

## Data Availability

The raw data of the studies are publicly available at ENA (European Nucleotide Archive https://www.ebi.ac.uk/ena/browser/) under accessions PRJEB21196 and PRJEB47555. The preprocessed data used for the demonstration run (OTUs table, OTUs Tree and mapping file are available at the tools github page: https://github.com/Lagkouvardos/Cronos/tree/main/Cronos_example.
