# Supplementary material for: Cronos: A Machine Learning Pipeline for Description and Predictive Modeling of Microbial Communities Over Time
Source: Front Bioinform. 2022 Aug 9;2:866902. doi: 10.3389/fbinf.2022.866902 (PMC9580867; doi:10.3389/fbinf.2022.866902)
Supplement: Supplementary file 1 [file Datasheet2.PDF]

Supplementary Material

1 SUPPLEMENTARY TABLES AND FIGURES

1.1 Figures

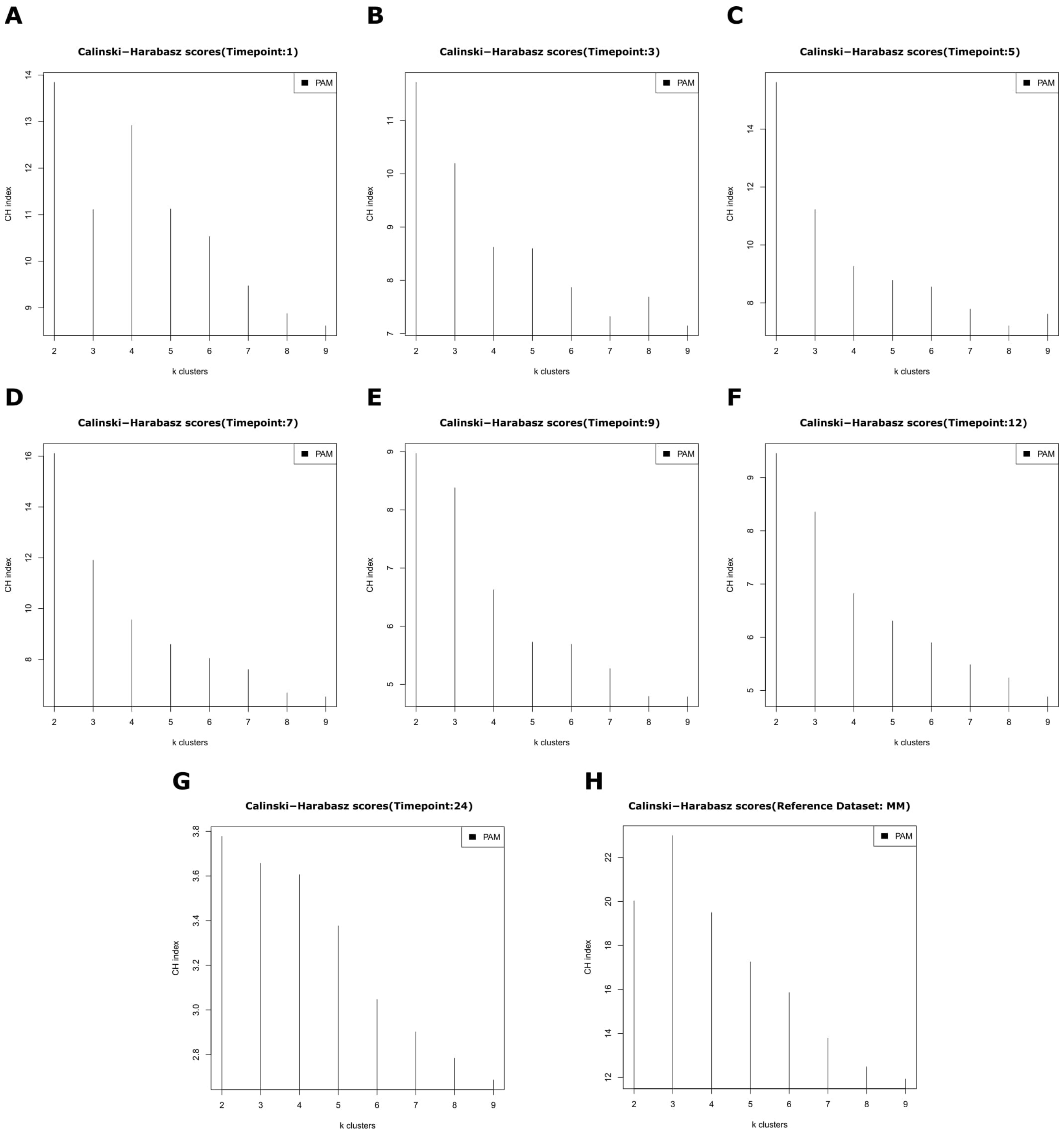

Figure S1. Calinski-Harabasz scores achieved for all timepoints and numbers of clusters. The number of clusters (k) is represented on the x axis, while Calinski-Harabasz scores are shown on the y axis.

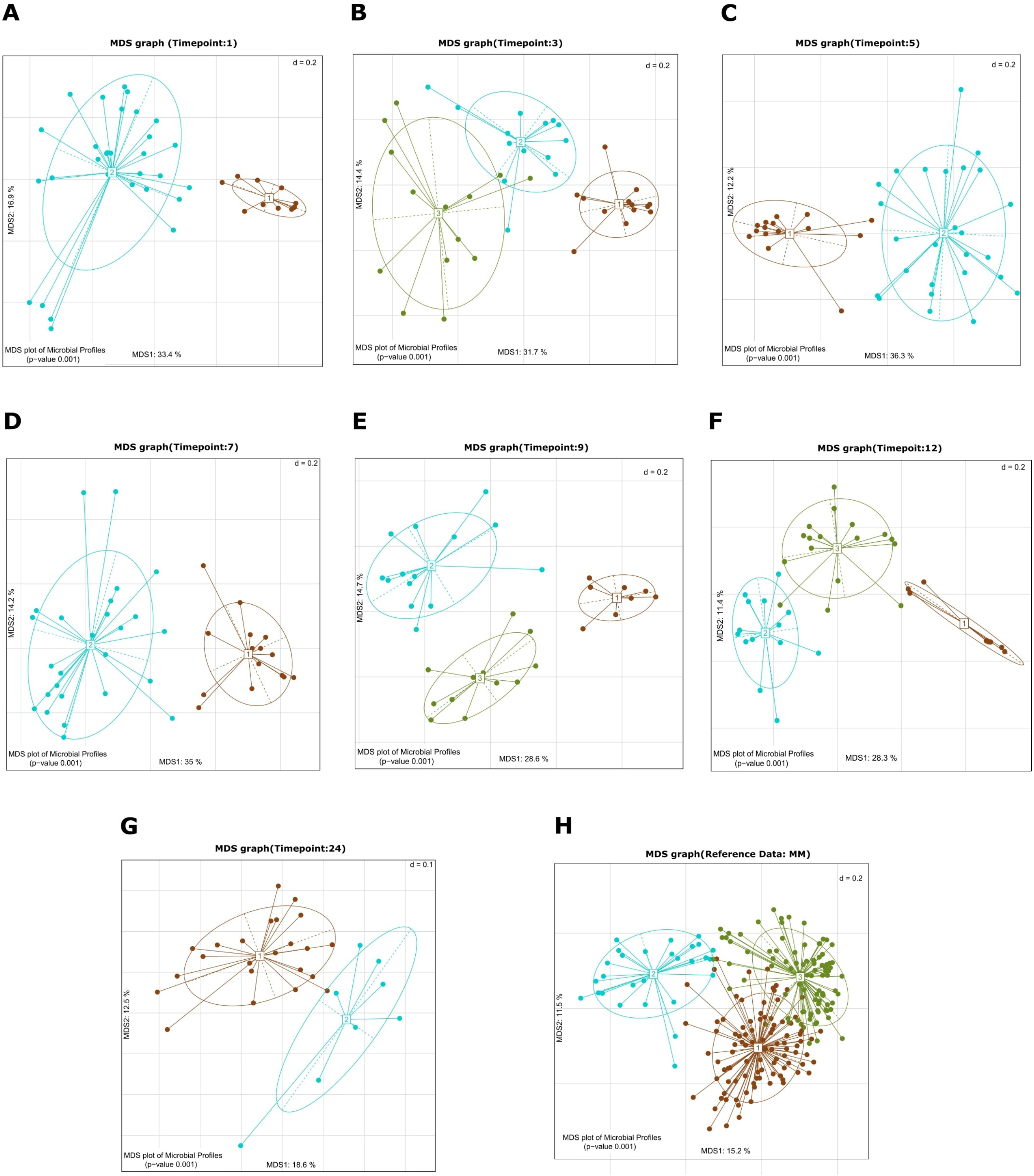

Figure S2. MDS plots of all time points. A,B,C,D,E,F and G represent 1,3,5,7,9,12 and 24 months of age, while H represents the external reference time point of students. Every dot is the projection of the relative position of the corresponding sample.

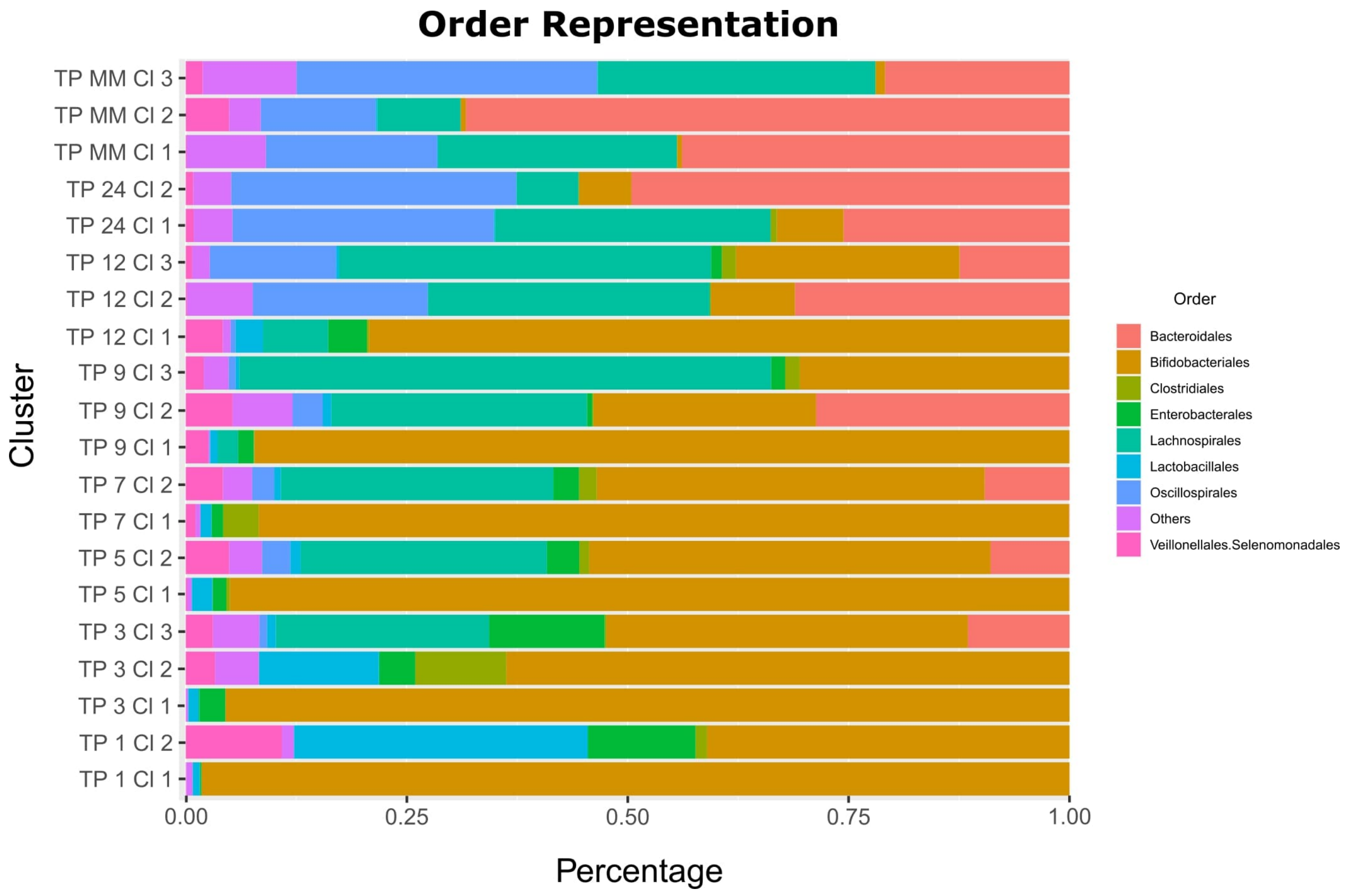

Figure S3. Order level taxonomic profiling of clusters of all time points. TP represents the time point and Cl the cluster. Every observed Order is denoted as a different color, while the x axis represents its relative abundance as ratio.

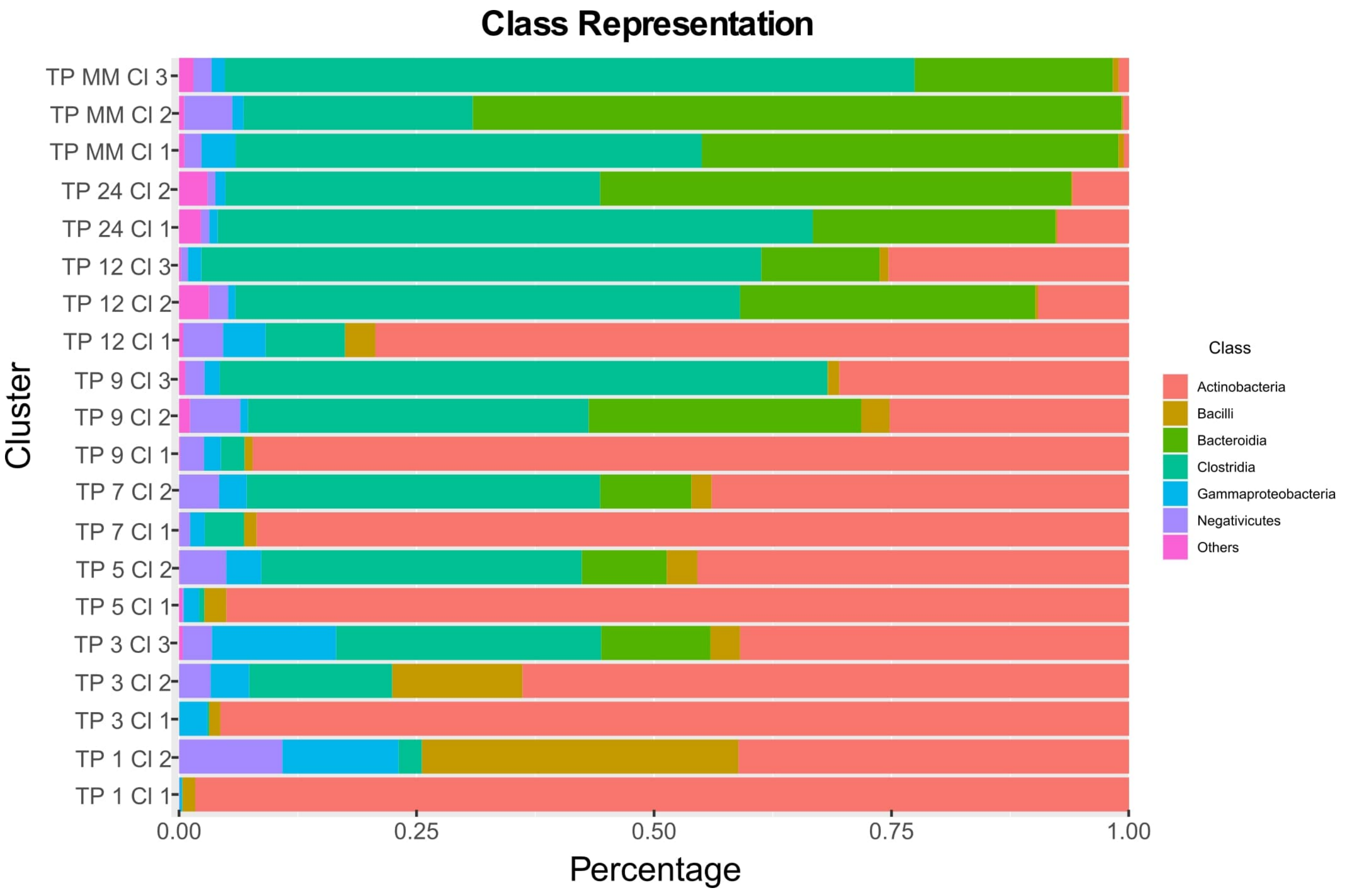

Figure S4. Class level taxonomic profiling of clusters of all time points. TP represents the time point and Cl the cluster. Every observed Class is denoted as a different color, while the x axis represents its relative abundance as a ratio.

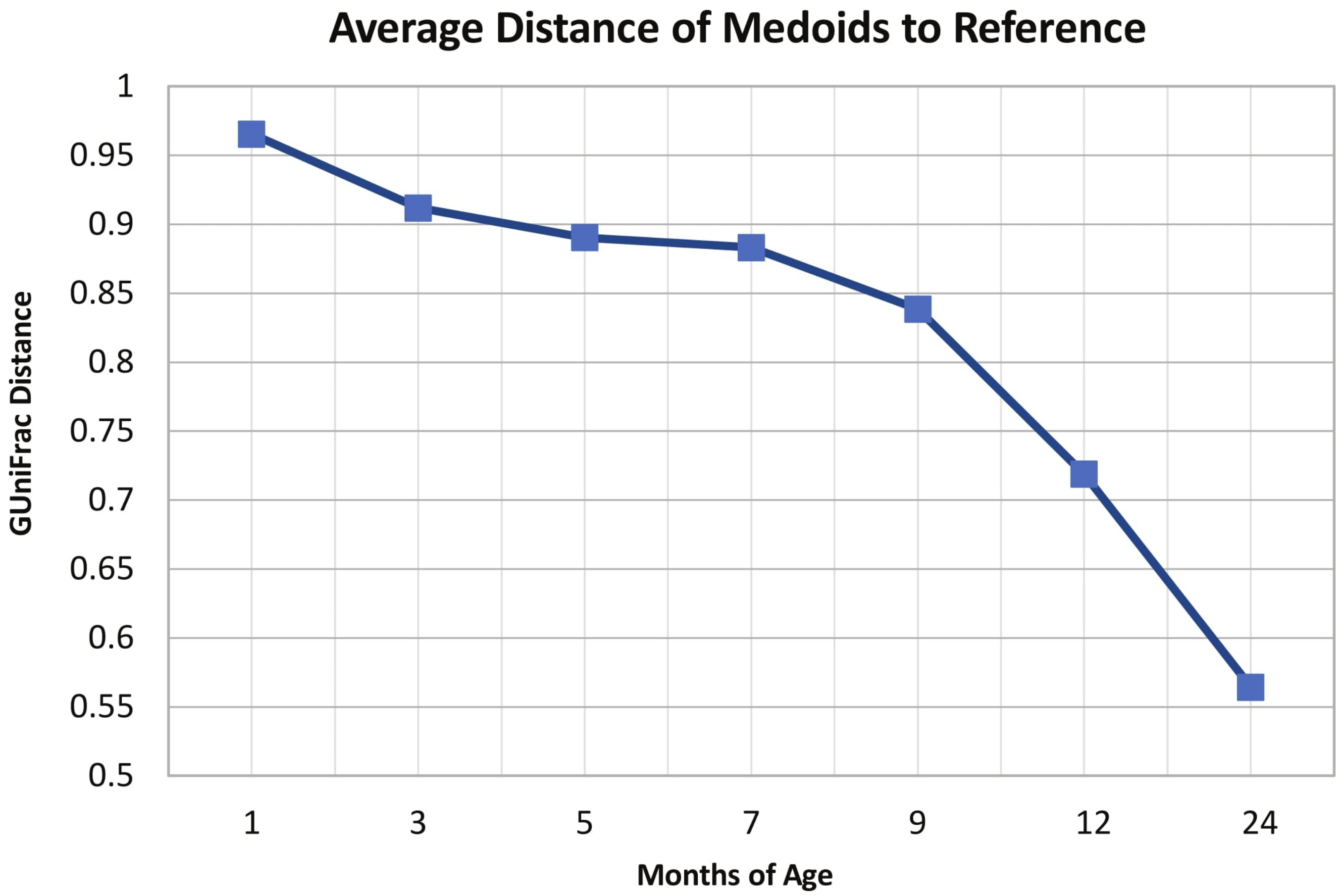

Figure S5. Average distance of the infants medoids at different months of age from the adult reference medoids. Distance was extracted from the GUniFrac dissimilarity matrix. The microbial profiles in early life appear distant following a rapid conversion towards the adult profile after the 9th month of age.
